# Time in ICU and post-intensive care syndrome: how long is long enough?

**DOI:** 10.1186/s13054-024-04812-7

**Published:** 2024-01-23

**Authors:** Dylan Flaws, John F. Fraser, Kevin Laupland, Jayshree Lavana, Sue Patterson, Alexis Tabah, Oystein Tronstad, Mahesh Ramanan

**Affiliations:** 1 Department of Mental Health, Metro North Mental Health, Caboolture Hospital, Caboolture, QLD Australia; 2 Critical Care Research Group, Adult Intensive Care Service, The Prince Charles Hospital, Brisbane, QLD Australia; 3 School of Clinical Sciences, Queensland University of Technology, Brisbane, QLD Australia; 4 Northside Medical School, University of Queensland, The Prince Charles Hospital, Brisbane, QLD Australia; 5 Department of Intensive Care, Intensive Care Unit, Royal Brisbane and Women’s Hospital, Brisbane, QLD Australia; 6 Department of Intensive Care, Adult Intensive Care Services, The Prince Charles Hospital, Brisbane, QLD Australia; 7 School of Dentistry, University of Queensland, Brisbane, QLD Australia; 8 Department of Intensive Care, Intensive Care Unit, Redcliffe Hospital, Brisbane, QLD Australia; 9 Department of Intensive Care, Intensive Care Unit, Caboolture Hospital, Brisbane, QLD Australia; 10 School of Medicine, University of Queensland, Brisbane, QLD Australia; 11 Physiotherapy Department, The Prince Charles Hospital, Brisbane, QLD Australia; 12 Critical Care Division, The George Institute for Global Health, University of New South Wales, Sydney, NSW Australia

## Abstract

**Background:**

Our understanding of post-ICU recovery is influenced by which patients are selected to study and treat. Many studies currently list an ICU length of stay of at least 24, 48, or 72 h as an inclusion criterion. This may be driven by established evidence that prolonged time in an ICU bed and prolonged ventilation can complicate post-ICU rehabilitation. However, recovery after short ICU stays still needs to be explored.

**Methods:**

This is a secondary analysis from the tracking outcomes post-intensive care (TOPIC) study. One hundred and thirty-two participants were assessed 6-months post-ICU discharge using standardised and validated self-report tools for physical function, cognitive function, anxiety, depression and post-traumatic stress disorder (with clinically significant impairment on any tool being considered a complicated recovery). Routinely collected data relating to the ICU stay were retrospectively accessed, including length of stay and duration of mechanical ventilation. Patients with short ICU stays were intentionally included, with 77 (58%) participants having an ICU length of stay < 72 h.

**Results:**

Of 132 participants, 40 (30%) had at least one identified post-ICU impairment 6 months after leaving ICU, 22 (17%) of whom had an ICU length of stay < 72 h.

**Conclusion:**

Many patients with an ICU length of stay < 72 h are reporting post-ICU impairment 6 months after leaving ICU. This is a population often excluded from studies and interventions. Future research should further explore post-ICU impairment among shorter stays.

**Supplementary Information:**

The online version contains supplementary material available at 10.1186/s13054-024-04812-7.

## Background

With most patients now surviving ICU, academic and clinical focus has shifted to quality of survival. Up to 80% of patients discharged from ICU experience ongoing cognitive, psychological, or physical impairments, known as post-intensive care syndrome (PICS), which can persist for years at great cost to patients, families, and society [1]. Despite over a decade of research, the aetiology and best prevention/treatment strategies for PICS remains elusive and an ongoing priority [1]. Optimising recovery is an ethical and economic imperative.

Two key challenges need to be overcome to support evidence-based prevention and treatment strategies [2] to improve outcomes. The first is identification of the characteristics of ICU patients who develop PICS, including modifiable and unmodifiable risk factors [3]. The second is to elucidate the ways which those factors complicate recovery.

Because PICS is a complex problem experienced by a heterogeneous population, our understanding is inherently shaped by which patients we choose to study and treat [4]. A recent systematic review identified that in nearly half of pertinent studies an ICU length of stay (LoS) of at least 24, 48, or 72 h was inclusion criteria [3]. The implication is that the risk of PICS among short ICU stays is low enough that study or intervention is unnecessary. This may be driven by established evidence that prolonged time in an ICU bed and prolonged ventilation can complicate post-ICU rehabilitation [5].

However, while patients with prolonged ICU stays may be at elevated risk of PICS, the recovery after short ICU stays still needs to be explored unless evidence confirms there is no burden of PICS among them.

This manuscript is grounded in a secondary analysis from the tracking outcomes post-intensive care (TOPIC) study [6], a prospective observational study that recruited participants discharged from participating ICUs. One hundred and thirty-two participants were assessed 6-month post-ICU discharge for impairments using standardised and validated self-report tools. These included measures of Physical Impairment (EQ-5D-5L [7]), Cognitive Impairment (PROMIS-Cog-8a [8]), Anxiety and Depression (Hospital Anxiety and Depression Scale (HADS [9])) and PTSD (Trauma Screening Questionnaire (TSQ [10])).

## Results

TOPIC included 132 patients. Thirty percent (*n* = 40) had at least one identified post-ICU impairment 6 months after leaving ICU, including 29% (*n* = 22/77) with LoS < 72 h and 33% (*n* = 18/55) with LoS ≥ 72h.

**Fig. 1:**
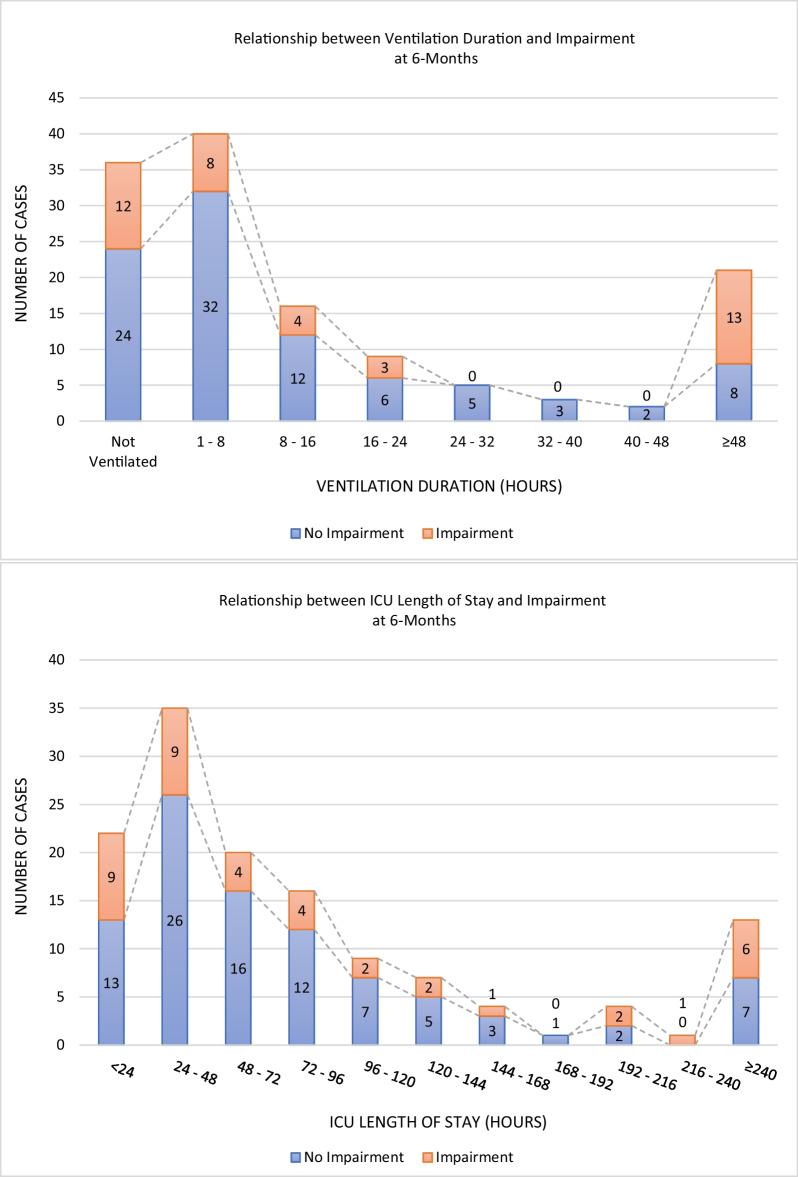
Relationship of ICU admission and ventilation durations and post-ICU impairment

Figure 1 shows the distribution of participants experiencing impairments by their ICU length of stay and ventilation duration, respectively.

Impairments were reported across the ICU duration of stay spectrum. The prevalence of physical, cognitive, and psychological impairments among the < 72 h LoS group was 14% (*n* = 11/77), 13% (*n* = 10/77), and 21% (*n* = 16/77), respectively. The ≥ 72 h LoS group had a comparable prevalence of physical impairment, with 19% (*n* = 10/54), a much lower cognitive impairment prevalence of 4% (*n* = 2/54) and slightly higher prevalence of psychological impairment, with 28% (*n* = 15/54).

## Discussion

Many patients discharged from ICUs after a short length of stay, and brief periods of mechanical ventilation go on to experience post-ICU impairments.

This population represents a substantial burden of morbidity. Over 188,000 patients were discharged from Australian ICUs in 2023, with a median ICU length of stay of 1.8 days [11], meaning over 50% were in ICU for < 48 h, a threshold below which many post-ICU studies and services are currently not recruiting. Our data suggest as many as 29,000 such patients may develop impairments in Australia each year and are currently being excluded from many studies and follow-up services.

These findings do not suggest that a long length of stay or ventilation duration is not an important risk factor for PICS, and it remains likely that prolonged ICU stays and associated factors such as patient characteristics, severity of illness, ICU delirium, and loss of muscle mass are important factors for clinicians to consider and address [1, 5], but this cannot be allowed to overshadow the morbidity faced by patients with shorter ICU stays.

The factors contributing to PICS among shorter ICU stays are likely to be different. These patients receive a lower dose exposure to in-ICU factors such as immobilisation and ICU delirium. Pre-ICU factors around how the illness/injury developed, within-ICU factors that are not time-sensitive (such as medications and procedures administered) and post-ICU factors such as external support systems are possible contributors to post-ICU impairment in this group. For patients that develop PTSD, it is important to consider that the index traumatic experience can have a very short duration (for example, a motor vehicle collision may only last a few seconds). As such, precipitants to PTSD may not show a temporal dose–response. Further, it is important to consider that such processes would not be limited to short ICU stay patients and may be represented across the ICU length of stay spectrum.

The prevalence of PICS among patients with a short ICU length of stay and ventilation duration in our study was considerable and represents a potential burden of morbidity that may be currently understudied and undertreated. It is therefore an urgent ethical and economic imperative that patients discharged after shorter ventilation and ICU admission times be included in both future research and treatment efforts.

### Supplementary Information

**Additional file 1.** Data used in analysis.

## Data Availability

Data are provided within the manuscript or Additional files 1.
